# Still rethinking external assistance for health

**DOI:** 10.1093/heapol/czad103

**Published:** 2024-01-23

**Authors:** Susan P Sparkes, Zubin Cyrus Shroff, Kara Hanson

**Affiliations:** Alliance for Health Policy and Systems Research, World Health Organization, Geneva 1211, Switzerland; Department of Health Financing and Economics, World Health Organization, Geneva 1211, Switzerland; London School of Hygiene and Tropical Medicine, London WC1E 7HT, United Kingdom

**Keywords:** health systems, health financing, external assistance, health policy

## Motivation

As we revisited our April 2022 announcement of this special issue on *Rethinking External Assistance for Health,* we were struck by the increasing relevance of the themes that were raised (Shroff *et al.*, 2022; HPP call for abstracts 2022). We are no longer speaking about the *potential* effects of the economic crisis triggered by COVID-19, but rather how countries and households are now experiencing the realities of fiscal tightening, increased sovereign debt, inflation and shifting geopolitics (International Monetary Fund, 2023).

Reflecting these pressures and changing dynamics, questions around the functions of external assistance for health and the forms it takes are high on the agendas of both donors and recipient countries. Structural issues around the long-term sustainability of donor-supported expansions in service coverage have been elevated. For example, the Future of Global Health Initiatives process is galvanizing voices of Southern stakeholders, together with donors and technical partners, to call for shifts in how external assistance for health is operationalized to support sustainable, country-led system strengthening efforts (Future of Global Health Initiatives, 2023). The ‘decolonizing global health’ movement (Khan *et al.*, 2021) challenges us to consider the rationale for external assistance and how it is delivered, arguing that aid should support and complement national priorities, not those set in Washington or Geneva (Drake *et al.*, 2023). Considering power dynamics and representation within the governance of external assistance for health forces questions about its future role and the form it should take, alongside deeper understanding of how historical context shapes current systems and dynamics.

There is clear urgency behind these questions as we see the world off-track to meet most of the health-related Sustainable Development Goals (SDGs) (World Health Organization, 2023). The recently released Universal Health Coverage Global Monitoring Report 2023 underscores this lack of progress, showing that service coverage improvements have stagnated since 2015 and the proportion of people facing catastrophic levels of out-of-pocket health spending continued to increase through 2019 (World Health Organization and International Bank for Reconstruction and Development/The World Bank, 2023), even before the impacts of COVID-19 began to be felt. This lack of progress is seen despite vast increases in external assistance for health between 2000 and 2020 (World Health Organization, 2022), in particular for disease-specific areas (World Health Organization, 2021b). These worrying trends coupled with larger global macroeconomic and social forces raise several questions about the future of external assistance for health.

## Still rethinking

The 13 research articles, innovation and practice reports and commentaries presented in this supplement provide concrete examples, ideas and reflections that add to our understanding of external assistance for health. They are timely, evidence-informed and relevant to both country- and global-level agendas. They build on an already-strong foundation of evidence and policies around the issues of alignment, country-driven approaches and de-verticalization (Oliveira‐cruz *et al.*, 2003).

The articles presented in this supplement dig into the structural and political underpinnings of external assistance for health that affect its effectiveness in supporting sustainable outcomes. In doing so, they provide important signposts in terms of best practices and lessons learned from problem areas. They present new, carefully researched experiences in transitioning away from external assistance for health, together with explicit analysis of the politics of external assistance for health, at both the country and donor levels. While adding to the evidence base, important gaps remain in terms of how to reform external assistance for health. In particular, there is a need for further research to understand how to: (i) make donors accountable to the ultimate beneficiaries of external assistance; (ii) develop viable strategies to increase domestic investments for health; (iii) overcome entrenched interests at both the donor and country levels that may be resistant to change in the face of demographic, epidemiological and economic shifts; and (iv) understand and differentiate between ‘global’ and ‘local’ representatives of global agencies; as well as many other areas.

## Key lessons

Despite the wide range of subject areas, three themes emerge from the body of knowledge presented in this supplement: (i) global shifts in the donor landscape, (ii) country-level engagement and political dynamics and (iii) how to structure external assistance to promote sustainable coverage.

With respect to global shifts in the donor landscape, the papers highlight three messages. The first is the need for external assistance to shift from potentially displacing domestic, public funds for basic service provision, including commodities and health worker salaries, towards a greater focus on investments in Common Goods for Health, such as surveillance systems and support for developing effective regulations and guidelines (World Health Organization, 2021a). This is highlighted in the paper by Nonvignon *et al.* (2024) that proposes a ‘New Public Health Order for Africa’, and in the paper by Tediosi *et al.* (2024) that seeks to identify appropriate strategies to transition away from external assistance for polio. The second is the value of technical support contributing to strong in-country technical capacities, illustrated in the paper by Huang *et al.* (2024) on Georgia and China, as well as of prioritizing South–South peer-to-peer learning through networks such as the Joint Learning Network on Universal Health Coverage examined in the paper by Hashiguchi *et al*. (2024). This is a marked departure from traditional models of technical assistance that prioritize knowledge emanating from Northern institutions and delivered by Northern experts through short-term missions and projects. The third message is the increasing importance of new actors in the donor space, most notably China, with the paper by Guo *et al*. (2024) illustrating how the evolution of Chinese external assistance for health has been directly linked to the country’s geopolitical aspirations and strategic support to fill gaps where other donors have often played a minor role.

Supplement papers also provide four lessons around country-level donor engagement and politics. First, fostering country ownership requires genuinely empowering national actors to decide what is supported through external assistance, who this support is targeted at and how this support is provided (Perera *et al.*, 2024, Shroff *et al.*, 2024). Second, purely technocratic approaches such as budgeting tools to bring about donor alignment and coordination will have limited effect unless power asymmetries and donor accountability to recipient countries are addressed (Sharma *et al.*, 2024). Third, trust between donors and national stakeholders and joint decision-making are vital enablers of sustained coverage post-transition, and lack of trust has negative implications for health (Shroff *et al.*, 2024; Zakumumpa *et al*., 2024). Fourth, entrenched domestic political interests (including within Ministries of Health) can play a major role in resistance to integration and programme alignment, including incorporation of donor-supported services into service packages (Neel *et al.*, 2024; Perera *et al.*, 2024).

The supplement provides three clear lessons on how the design of external assistance can promote sustained coverage. The first is the need for external assistance to be more flexibly adapted to different contexts, including small island states that may not be able to benefit from economies of scale (Meessen *et al.*, 2024). This flexibility extends to measures used to initiate donor transitions, with calls to replace single indicators such as Gross National Income per capita with more comprehensive measures that provide a holistic understanding of the vulnerability of individual countries to shocks and fiscal space considerations (Kim *et al.*, 2024; Meessen *et al.*, 2024). The second is the value of channelling donor funds through domestic, public financial management systems, as well as clear domestic co-financing policies. This transparency and buy-in from the domestic, public financing system can also enable a smoother transition process to ensure countries are capacitated to sustainably and efficiently finance previously donor-supported services (Shroff *et al.*, 2024; Zakumumpa *et al*., 2024). The third is the importance for donor funding to not duplicate existing, domestic structures and functions, in particular around inputs such as human resources for health, medicines and supply chains or infrastructure. The papers clearly demonstrate the critical role of aligning with national structures and practices in enabling sustained intervention coverage post transition (Perera *et al.*, 2024; Shroff *et al.*, 2024; Zakumumpa *et al*., 2024).

## Concluding message

The call for papers for this supplement elicited a huge response from the research and policy communities, indicating the continued salience of the topic of external assistance for health. The challenges of encouraging a re-balancing of power in how development assistance is structured and delivered while simultaneously maintaining the commitments of richer countries in the face of growing domestic fiscal and political pressures are real. Yet, strengthening this compact will be necessary for continued progress towards the SDGs in face of the polycrisis the world faces. Health Policy and Systems Research’s emphasis on country ownership, context, policy relevance and squarely addressing issues of equity and power make it ideally suited to inform the further thinking and action that are needed.
